# Shared Decision Making in Psychiatry: Dissolving the Responsibility Problem

**DOI:** 10.1007/s10728-022-00451-7

**Published:** 2022-12-03

**Authors:** Leila El-Alti

**Affiliations:** 1 School of Health and Social Care, Edinburgh Napier University, Sighthill Court, EH11 4BN Edinburgh, UK; 2 Department of Philosophy, Linguistics, and Theory of Science, University of Gothenburg, Box 200, 405 30 Gothenburg, Sweden

**Keywords:** healthcare ethics, mental illness, person centered care, patient responsibility, patient emancipation

## Abstract

Person centered care (PCC) invites ideas of shared responsibility as a direct result of its shared decision making (SDM) process. The intersection of PCC and psychiatric contexts brings about what I refer to as *the responsibility problem*, which seemingly arises when SDM is applied in psychiatric settings due to (1) patients’ potentially diminished capacities for responsibility, (2) tension prompted by professional reasons for and against sharing responsibility with patients, as well as (3) the responsibility/blame dilemma. This paper aims to do away with the responsibility problem through arguing for a functional approach to mental illness, a blameless responsibility ascription to the person with mental illness, as well as a nuanced understanding of SDM as part of an emancipation-oriented PCC model.

## Introduction

Person-centered care (PCC) has recently risen as a celebrated model of healthcare delivery which places a person at the center of the care [1] and individualizes the care to better fit that person’s life. PCC pushes away from the biomedical model of care and represents a holistic, non-reductionist view of the complex ‘person’ rather than the passive ‘patient’ [1–5]. Because of its focus on shared decision making (SDM) and the importance of patient narrative, PCC becomes more oriented toward emancipation of the person rather than typically desired medical outcomes [6, 7]. Therefore, PCC is often presented as a paradigm shift [8, 9] taking place through transfer of power and, consequently, responsibility from a healthcare professional (HCP) to a receiver of care [5, 8, 9]. SDM thus becomes the part of PCC framework in which a partnership between the HCP and patient is expected to emerge [2, 8] in order to realize an emancipatory outcome [6]. Based on the patient narrative [10], this dialogue between the HCP and patient might include instances of disagreement and debate [11] or consensus and compromise [12] to reach a plan of care tailor-made for the individual person.

Since paternalism has traditionally been the dominant perspective within psychiatry [13], employing a PCC framework in psychiatric contexts brings about a complex responsibility problem which has led to some reluctance within psychiatry to fully embrace PCC [14]. This problem consists of a combination of three difficult and distinct^1^ challenges relating to patient responsibility: whether persons with mental illness are capable of taking responsibility, how HCPs can share responsibility with them, and a responsibility/blame dilemma. This paper neither presents a new account of responsibility in mental illness nor does it aim to distinguish between different kinds of capacities in different disorders and how they relate to PCC. The aim is to dissolve the concerns surrounding the participation of mentally ill persons in SDM as a part of a PCC framework of care, as said concerns frequently appear to be in opposition to a person-centered psychiatric practice. To do away with this responsibility problem I will argue, first, that ascribing and sharing responsibility is theoretically feasible in psychiatry if we develop a nuanced understanding of SDM as a part of the emancipation-oriented PCC model, adopt a functional approach to mental illness, and practice responsibility without blame.

In the next section, I will explicate the responsibility problem as arising from the intersection of SDM with psychiatry. In section III, I will dissolve the first point of the responsibility problem, i.e., the *capacity question*, after presenting a detailed description of SDM and its components. In section IV, I will introduce the functional approach to address the tension surrounding the how of *responsibility ascription*. Section V will tackle the *blame dilemma* through discussing Hanna Pickard’s account of blameless responsibility. I will consider in section VI whether there are instances when SDM is infeasible in psychiatry. The conclusion will provide a brief summary of arguments.

## The Responsibility Problem

The idea of shared responsibility becomes an immediate byproduct of SDM, when the person is expected to be a partner in the decision-making process and uphold her end of the bargain through executing the plan of care on which she agreed with the HCP. This shared responsibility^2^ stems from the professional-patient partnership and the deliberation taking place in SDM [15]. The implicit assumption about the person involved in SDM is thus that she possesses a capacity for responsibility requisite for active participation in such a process.

This expected upshot of emancipation of persons in healthcare suddenly becomes problematic if PCC is to be applied in areas such as psychiatry, where sharing responsibility with a patient is potentially challenging due to her capacities being affected by mental illness(es). Consider a person living with schizophrenia whose delusions, for example, affect her understanding of certain aspects of reality, her insight, decision making, and executive capacity. All of these capacities *seem* to be indispensable for successful SDM, not only for the patient’s active participation in SDM, but perhaps also for how responsibility is to be ascribed.

The *responsibility ascription* question arises because, as the aim of PCC is empowerment, it becomes essential in a psychiatric setting for the care to provide patients with needed opportunities to act on their own accord thus, becoming (more) capable as well as (more) responsible decision makers^3^. On the other hand, HCPs also risk harming patients whose decision-making capacity is fragile by overburdening them with the responsibility for health-related decisions or tasks at which they will probably fail due to the nature of their illness. This means psychiatric HCPs would have reasons to simultaneously share as well as withhold responsibility.

To further complicate matters, as responsibility in SDM is shared, it becomes implicit that patients who do not fulfill their part of the agreement are blameworthy for not following a plan of care to which they agreed. At the same time, HCPs blaming patients is not an ideal method to maintain a good professional-patient relationship, as it creates a hostile atmosphere where the patient fears the reaction of the HCP. While this *blame dilemma* is relevant in all PCC settings [10], it is especially problematic in psychiatric settings where mental illness can affect the person’s ability to control her actions^4^ and may consequently lead to more blame from the HCP. Such blame may not only seem inappropriate, but outright harmful and counterproductive to the aims of the care [18]. Therefore, the question of patient responsibility becomes crucial if we are to think of SDM in a psychiatric healthcare context, as it has implications on whether the idea of person-centered psychiatric care is plausible.

## SDM and Patient Capacities

PCC as conceptualized by El-Alti et al. [6] is a healthcare model with “three interconnected levels” (p. 47). The model’s *base* consists of a core assumption about the complexity of the individual person, its *action* level involves a partnership between the HCP and patient through SDM, and its *purpose* is patient empowerment. Being the central process in the PCC action phase, SDM recognizes the authority of two parties on extreme ends of a spectrum. It is the meeting point between a professional’s paternalism and expertise on one side, and a patient’s autonomy and narrative on the other [18, 19]. Sandman and Munthe describe SDM’s nine different versions or levels, starting with “patient adapted paternalism” (p. 291) during which a patient shares information about herself but the final care decision is made by the HCP, and ending with “professionally driven best interest compromise” (p. 292) during which compromise might follow from deliberation and conflict of the two parties^5^ [12].

If we are to visualize a continuum of medical decision making with one end representing extreme paternalism whereby HCPs make all decisions regardless of patient wishes, and another end being absolute patient autonomy whereby the patient demands what she pleases from the HCP; then SDM would fall right in the middle. If the nine levels presented by Sandman and Munthe [12] are adapted into this visualization^6^, then the highest level of SDM would be the midpoint on the continuum as it involves the most deliberation, conflict, and compromise between the HCP and patient.

**Fig. 1:**
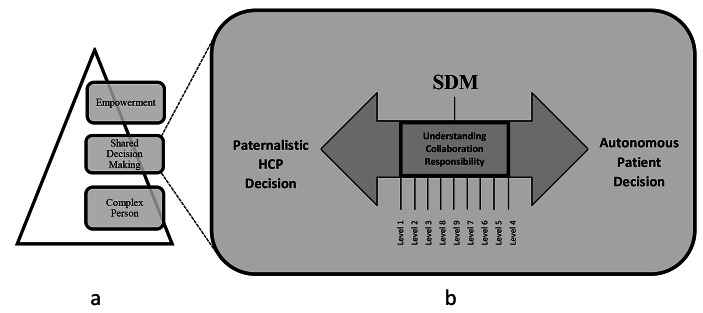
(a) Three levels of PCC adapted from El-Alti et al. [6] (b) Essential SDM elements illustrated as a continuum of different levels between two extreme ends of paternalistic and autonomus decision-making. The nine levels of SDM are adapted from Sandman and Munthe [12]

**Fig. 2:**
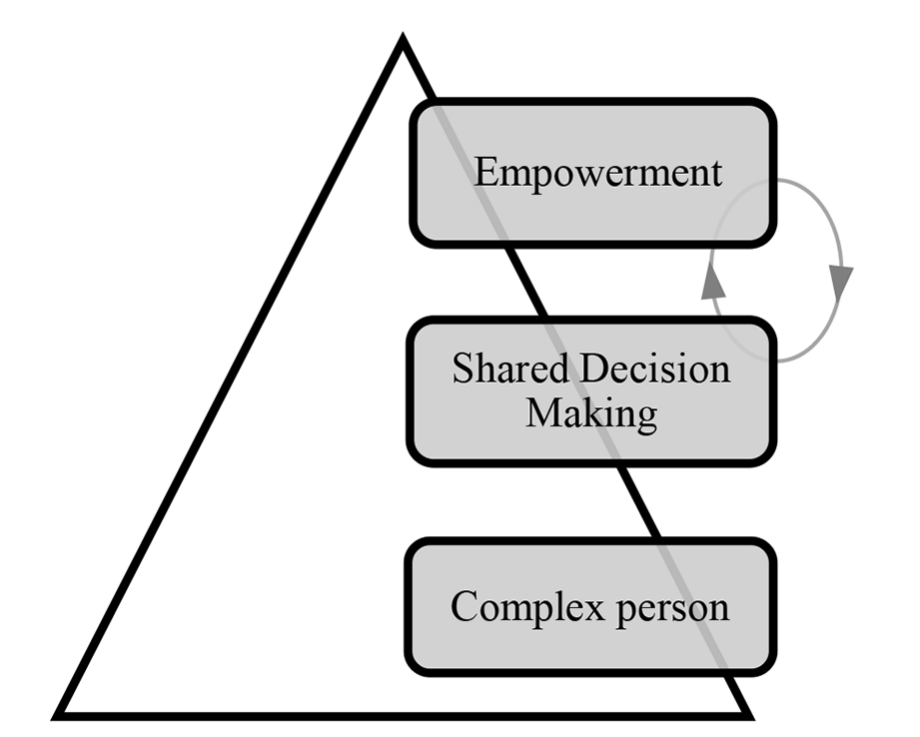
The three levels of PCC as adapted from El-Alti et al. [6] showing a positive feedback loop between empowerment and SDM

The SDM thus identified requires a reciprocal deliberative interaction to take place between the deciding parties, which seemingly assumes a set of capacities and executive abilities in each party. In this SDM, the patient cultivates understanding of basic aspects about her condition as well as facts about treatment options and related decisions. In a reciprocal manner, the HCP cultivates understanding of the patient’s story and needs as part of her narrative. The two parties then collaborate to find a decision that would satisfy them both. Finally, the resulting (shared) decision expresses the shift of power accomplished through understanding and collaboration, which in turn results in a shift of responsibility to be then shared between the two partners, as previously discussed. Therefore, a person partaking in SDM *understands* relevant aspects of her illness and possible treatment, *collaborates* with the HCP, and *takes responsibility* in relation to ensuing care decisions^7^. Please refer to Figs. 1 and 2.

In a standard non-psychiatric medical setting, where adult patients are presumed to live with no mental illness, the responsibility problem of SDM is not expected to surface, as the patients’ mental capacities are not usually under question. This means that the responsibility problem seems to come to light exclusively in contexts where a person’s capacities are assumed to be atypical^8^^,^^9^. Hence, the *capacity question* in the responsibility problem appears to be a rather intuitive concern for SDM practice, arising due to a tense marriage between high SDM demands and questionable patient capacities. It appears reasonable to ask whether mental illness affects a person’s abilities to a sufficient degree such that she can no longer keep up with SDM demands.

However, while this concern *seems* intuitive, it represents a misconstrual of the PCC model and its SDM process in two ways. First, it conflates the three SDM components with demands or requisite criteria for participation, such that if a person is unable to fulfill (all of) them she risks being disqualified from the process. More importantly, it makes participation in SDM, and by extension PCC, contingent on certain personal characteristics, viz. a person has to possess certain abilities in order to fit the process. Both these ideas are in direct opposition of the PCC model described at the beginning of this paper.

Although the responsibility problem stems from the *action* level of PCC, i.e., SDM [6], the latter does not take place in isolation from the rest of the framework of which it is an essential part. PCC rests on the idea of the complexity and uniqueness of the person, respects her governance over her own life, and opposes the one-size-fits-all approach through individualizing care for every person [6]. Implicit through asking whether certain people are suited for PCC is an assumption that PCC has set criteria to be met, thereby attaching standardized conditions to a model which opposes standardization. Hence, from a PCC perspective, it is contradictory to ask whether a certain person fits a model which itself is defined by personalizing the care in order to fit that person. It is irrelevant to PCC the category of illness under which a person falls or which (dis)abilities she has. Rather than being requirements to be met, the three SDM components are to be understood as guidelines which are personalizable to whatever capacities a person possesses. The nine levels of SDM provide a theoretical glimpse of the pliability of the actual process, whereby an appropriate SDM level can be employed based on the person’s capacity and can shift over time depending on her progress or relapse.

This is not dissimilar to choosing a book for someone based on her age, reading ability, preferred language, interest, education level, etc. One’s choice of book will likely differ among different people as well as change over time for one person based on the perceived level of difficulty of the book, new interests, and other considerations. This analogy also helps demonstrate how a book is chosen to suit a person’s needs and abilities, instead of selecting the book in advance and then assessing a person’s ability to read it^10^.

## The Functional Approach

Granted that the *capacity question* has been dissolved in the previous section through a nuanced understanding of PCC as a model which fits the person rather than the opposite, the responsibility problem’s second question vis-à-vis *responsibility ascription* might be trickier to address. One of the reasons for this difficulty is the ubiquitous image of the person with mental illness as unpredictable, untrustworthy, aggressive, or lacking control as often perceived by the general public [21] and perpetuated by mainstream media [22], philosophers [23], or HCPs [24, 25]. The systematic psychiatric classification and description of mental illnesses and their symptoms also reinforces a stagnant quality to persons living under the label of their diagnoses. Persons with mental illness often face negative attitudes, unfounded stereotypical assumptions, fixed assessment of (in)capacities based on diagnosis, labelling, and stigma by psychiatric and non-psychiatric HCPs, which potentially contributes to poor psychological and physical health outcomes [26–29].

Another reason for the difficulty lies in the absence of one account of responsibility which could practically ease the tension surrounding how responsibility can be ascribed to persons with mental illness. There is neither consensus on accounts of responsibility in general, nor is there one regarding how to assign responsibility in certain mental illnesses accordingly [30]. Thoughts and actions affected by mental disorders vary so much that it is extremely difficult to have an account of responsibility encompassing the variabilities of all mental disorders, as much as it is meaningless to have different accounts for different disorders.

Even though the two questions overlap, it is important to note that the *responsibility ascription* question is not a question about a person’s capacity for responsibility, which has already been addressed in section III. Rather, this aspect of the responsibility problem concerns *how* HCPs (should) ascribe responsibility *based* on a person’s capacity, as there are reasons to both share and refrain from sharing responsibility with patients. Hence, what we are looking for is a practical way to ease the HCP’s burden of how to share responsibility with patients in SDM.

Mental illnesses are heterogeneous [31, 32] and exist on a spectrum [33]. Not only do two people with the same diagnosis vary in terms of symptoms and severity of illness but the illness itself also varies in the same person across time [34]. It has long been believed that schizophrenia, for instance, was a deteriorating condition yet many studies show that recovery is quite common [35, 36], and that it is completely possible to be diagnosed with schizophrenia without suffering from cognitive decline, apathy, or disorganization^11^ [37]. Therefore, any serious attempt to address the tension between the duty to protect a person with mental illness from being overburdened with responsibility and sharing responsibility with her when needed, must also take into consideration the person’s unique expression of an illness be it through time or in comparison to others.

When PCC rests on the idea of the uniqueness and complexity of a person and faces away from standardization, it does not come as a surprise that in order to make SDM fit persons with varying capacities we must adopt an approach which itself recognizes variability and opposes the status categorization of persons. In other words, we are required to shift the way we think about mental illness as something variable rather than static. In contrast to the *status* approach which is an overall perspective on a person experiencing symptoms across time, a *functional* approach [38] focuses on practically relevant symptoms which are subject to change depending on time and context. Aspects of functionality which are usually assessed can include simple tasks like self-care, cleaning or other chores, exercise, and social meetings; or more complex activities such as taking care of financials, planning and problem solving, coping with challenges, having a job, etc. [34].

The functional approach not only takes into consideration the variability of the person and her capacities, but also her unique expression of the illness. This is valuable for SDM because executional decisions take place in a specific moment in time which makes the person’s specific functionality at *that* instance, rather than in general, more relevant to the decision at hand. This shifts the weight typically placed on a diagnosis-dependent all-or-nothing *responsibility ascription*, i.e., a *status*, and emphasizes the variability of symptoms which could include temporary or permanent periods of mild or absent illness [37] as well as potential recovery [41–43].

The call for a functional approach does not intend to replace or eliminate the diagnostic categories of mental illness as it is pragmatically difficult to collapse said categories when they dictate, in many ways, various treatment options. And while having an anxiety disorder might appear markedly different from living with schizophrenia, the variability within the illnesses themselves across individuals and time as well as how that variability affects responsibility and decision making, make the classification unhelpful in terms of ascribing responsibility in SDM.

## The Blame Dilemma

Even if SDM practice, and its implied responsibility sharing with patients, is theoretically warranted in person-centered psychiatry, such a practice may raise objections about possible undesirable consequences for persons with mental illness. One specific risk has to do with the strong link between practices of attributing responsibility and practices of blaming people who fail to take their responsibility.

Consider the case of Annie, a person with no history of mental illness. During an argument, Annie gets angry after being called stupid and punches her friend in the face. Annie was aware of what she was doing and was not coerced to punch her friend, but rather got emotional and decided to resort to a violent action when she could have simply chosen not to do so. For example, she could have told her friend that she does not like being called stupid, walked away, or simply refrained from reacting at all. Because she chose to react violently knowing fully well that she will harm her friend, we tend to think that Annie’s anger does not provide an excuse for her action, and that she is responsible and deserves to be blamed for what she did.

This association of responsibility and blame gets more complicated when we consider the case of Zoe, a person diagnosed with an illness typically characterized by aggressive behavior and poor impulse control. Zoe is aware of her illness and its symptoms and wants to get better, but she struggles with anger almost every day. During a stay at the inpatient psychiatric ward, a nurse tells Zoe she should take her medication multiple times which makes Zoe angry and leads to her hitting the nurse with a chair. Even when her illness does not affect her understanding of the situation, Zoe’s poor impulse control affects the degree of control she has over her action and thus (to some degree) her responsibility for it. The question is whether this aspect of her mental illness provides a *sufficient* decrease of Zoe’s responsibility for her behavior to be *excused –* such that it would appear (entirely) inappropriate to blame her.

The reason why it is trickier to answer this question in Zoe’s case is that we tend to group responsibility and blame together. Our blame response seems to follow naturally once another person’s responsibility for a wrong action is established. It seems bizarre to even think about one without the other. When we are convinced that a person is responsible for knowingly committing a certain wrongdoing without a good excuse, then we usually blame her for that action. If two students agree to each complete a part of a joint project and they fail to pass the course because one of them did not submit the part she was responsible for, we tend to blame her for this outcome. However, if we find out that she is in the hospital after being in a car accident, we accept that as a sufficient excuse for her failure to submit her part of the project. In Zoe’s case, one could argue that because her illness is characterized by anger and impulsive behavior, she could be excused for hitting her nurse. It is not clear, though, whether excusing Zoe means that we only refrain from *blaming* her for hitting her nurse or whether it also implies that she is not at all *responsible* for that action.

The cases of Annie and Zoe consecutively illustrate a useful distinction presented by Hanna Pickard between cases of ‘can but does not’ versus ‘wants to but cannot’ [44]. She says there is a difference between being *able to refrain* from problematic behavior but *not doing so* sometimes, on one hand, and *wanting to refrain* from the same behavior but *not being able* to do so, on the other [44]. Based on this distinction, Pickard has outlined a pathway for how a practice of sharing responsibility in psychiatry need not be strongly linked to blame [23]. Because we are interested in determining the appropriateness of ascribing responsibility and blame in cases where there is strain on the ability to control actions, we will focus on the case of ‘wants to but cannot’, like in the example of Zoe.

Pickard’s blameless responsibility project advances three major points. First, mental illness is a restricting condition which limits a person’s capacity for control. Second, however, mental illness does not *extinguish* a person’s responsibility. Third, the typical blaming response which we often associate with wrongdoing is destructive to the person with mental illness and should be avoided. In order to address the *blame dilemma* in SDM, it is important to explain the grounds of Pickard’s third claim, and to outline how she thinks the first two claims provide a pathway for blameless responsibility^12^.

When we speak of responsibility we seem to distinguish between voluntary and involuntary behavior, with the voluntariness aspect being the choice and degree of control the person has [45]. However, choice and control in many cases of mental illness can be limited in comparison with the norm. Thus, even if Zoe had a choice to refrain from hitting her nurse, her capacity for control over that choice is reduced due to her illness. Nevertheless, her control is not eliminated altogether [23] but is constrained by the illness which in turn reduces her degree of responsibility. Yet, even when control is limited, a person is not stripped of all capacity for voluntary action and can still be attributed some responsibility for her actions [23]. In fact, in clinical psychiatric treatment the inherent assumption is that patients *are* capable of controlling their actions and can thus be asked to cease behaving in a certain way [23]. Attributing responsibility may thus serve a meaningful therapeutic purpose.

Blame, on the other hand, is a punishing mental state and the presence or absence of which depends on the change in attitude of the blamer as well as how this change is experienced by the target [46]. This affective response could be communicated through emotions like anger, resentment, contempt, indignation, disappointment, and/or through behavior targeting the blamed party like shunning, rejection, abandonment, criticism, and others. Another distinctive feature of blame is that the blamer feels *entitled* to having these negative emotions and attitudes [46]. But in a care situation, especially one where the patient suffers fragile capacities, blame is generally undesirable and potentially destructive. This relates not only to the health outcomes, but also to how the care affects patient capacities.

Therefore, when Pickard refers to blameless responsibility she means responsibility without affective blame, and hence without the sense of entitlement to communicate the negative attitudes which are likely to be therapeutically damaging [46]. This means that we hold a person to account for her wrongdoing without letting blame taint our emotions or messages. Responsibility without blame can be demonstrated through attitudes of mental HCPs in therapy and is provided a rationale through the duty of care [45]. Compassion and empathy are central to therapeutic care because they stand in opposition to the blaming response, as compassion seems to push away the negative emotions that are central for affective blame [46].

This is not the same as saying that HCPs should suspend their own moral judgments about the rightness or wrongness of their patients’ actions. Normative boundaries are not detachable from the therapy, as the mere fact that we try to change certain behaviors of patients implies that we have judged them to be wrong. What is practically meant by a blameless responsibility is that vocalizing normative thoughts when dealing with a patient can take place without using the emotionally charged language of blame, be it in words or actions.

The notion of blameless responsibility offers a pragmatic solution for the *blame dilemma* in SDM. In practice, sharing responsibility blamelessly in SDM would ensure a culture of compassion and respect, as well as a professional relationship between HCPs and patients while avoiding the described therapeutic pitfalls. This is achieved when (as in the example above) an HCP holds a patient responsible for her actions (including harmful ones), while suspending her own attitude of blame. Pickard regards this as the only practical way to change a patient’s problematic behavior, as doing so also prevents what Pickard refers to as the “rescue-blame trap” (p. 4) whereby in order to ensure a no-blame attitude, the HCP avoids holding the patient responsible [45]. From a PCC angle, the rescue-blame trap defeats the purpose of patient emancipation.

## When SDM is Impractical

One objection HCPs might put forth is that a person’s limited insight into her illness is a contraindication for the whole SDM process. Lack of insight is a common symptom of psychotic illnesses, for example, and holds a special importance for common psychiatric care strategies. It is often thought that patients who have more insight into their illnesses are more open to different treatment options and have better adherence, and some HCPs believe that the lack of insight is anathema to SDM [47]. In reality, the specific lack of insight linked to the disease is often irrelevant for patients’ ability to take part in treatment decisions or to follow them, as treatment options can often be phrased and discussed in terms of measures and symptoms of which the person is aware [48].

There are areas of capacity that are not affected and beliefs that are untouched by mental illness, and with the right support and guidance, a person can also learn to control behaviors and thoughts that *are* under the influence of the illness [23]. The mere presence of a mental disorder does not cancel the capacity for a person to give informed consent [37], have an understanding of (aspects of) one’s health situation, collaborate with her HCP, or take responsibility [30]. Even when a person has diminished capacity for something it is not equivalent to having no capacity for it at all [30].

Kleptomania, for example, affects a person’s ability to control behavior when it comes to compulsive stealing but has no effect on “acting out of aggression” (p. 21) [30]. Therefore, a person diagnosed with kleptomania can be held responsible for being aggressive. Analogously, a person diagnosed with schizophrenia who hears the voice of God and believes she is an angel in human form, can also be held responsible for behaviors unrelated to this conviction, such as being aggressive towards a fellow patient or an HCP. Even if the person’s delusions include a belief about a duty to rid certain people of their demonic possessions by hitting them on the head, she can still hold a concurrent belief that harming or killing others is wrong. This means that although her understanding about a certain *aspect* of reality is distorted, not *everything* about her understanding of reality is necessarily affected. This person can still be engaged in a discussion about the importance of treatment options, and be held responsible for her actions in light of that^13^. She can be supported to refrain from hitting others and reminded that the action constitutes harm. Thus, the insight objection is but a reiteration of the *capacity question* and a misunderstanding of the PCC model.

Another possible objection could be acute phases of illness during which a person becomes *temporarily* indisposed, and by virtue of the severity of the symptoms, unable to take part in any form of decision making or even less be ascribed responsibility. It is helpful to be reminded here that this is not unique to mental illness but holds true to any person who is in any kind of severe distress, be it physical or psychological. Anyone in severe physical pain, having a panic attack, or feeling faint due to hypoglycemia, for instance, is also temporarily incapable of taking part in SDM. This hypoglycemic person is as functionally incapacitated, from an SDM standpoint, as a person in an acute psychotic or catatonic phase.

There might still be, however, limits to how far a functional, blameless approach in SDM can reach. Persons who live with extreme intellectual disability or are so severely ill that their illness is completely resistant to all kinds of attempted treatments, will probably not benefit from a functional perspective, as their functionality is severely limited and might not improve over time. For the purpose of not further contributing to the perpetuation of stereotypical representation of mental illness through stressing *in*capacity rather than *variability*, worst case scenarios should not be generalized, as most persons, in fact, do not end up on the extreme end of the mental illness continuum [30] which would also make said generalization fallacious.

Not only is SDM an essential process in a model aiming to empower a person, but it can also be used as a tool to help improve a person’s capacities. Evidence today shows that cautiously applied SDM strategies may help improve patient outcomes and her decision-making capacities [49–51] to facilitate even more advanced SDM. For example, collaboration and SDM with persons suffering from psychosis has been shown to be a more effective treatment than directly jumping to anti-psychotic medication prescription [37, 52]. Other empirical studies have demonstrated that collaboration and training lead to positive patient results, such as increased desire for taking responsibility after SDM training [53]. In the same manner as one needs to actually ride a bike in order to learn how to successfully ride a bike, capacities such as the ability to make decisions and taking responsibility are not all-or-nothing type of capacities but ones which can be nurtured, trained, and developed over time. In that sense, empowerment through SDM becomes a means as well as an end for person-centered psychiatric care. Please refer to Fig. 2.

## Conclusion

In this paper, I have introduced a three-layered complex responsibility problem peculiar to the SDM process in psychiatric healthcare contexts. After presenting three SDM components, I argued that a nuanced understanding of PCC as an emancipation-oriented model revolving around the person and made to fit her, dissolves the question regarding ‘required’ capacities for SDM. I then argued that symptom variability of mental illness as well as PCC’s tendency for individualization justify the functional approach as a practical method to assign responsibility to patients. In order to address the *blame dilemma* in the responsibility problem, I employed Hanna Pickard’s blameless responsibility account which advances an empirically informed, more compassionate portrayal of the person with mental illness and endorses divorcing responsibility and affective blame in practice.

Whether stemming from a caring or paternalistic viewpoint, the assumption that SDM is infeasible due to acute illness episodes or lack of insight is inaccurate. There is evidence suggesting that SDM can actually benefit patients with psychiatric illnesses and improve their capacities over time. By dissolving the responsibility problem, I intended to show that SDM is (at least) theoretically feasible in psychiatric healthcare contexts. However, confirming the effectiveness and pragmatic feasibility of the functional approach combined with a blameless responsibility ascription to persons in person-centered psychiatric care settings will have to rest on the shoulders of empirical intervention studies.

## Footnotes

1 The three challenges of the responsibility problem are distinct, yet bearing interesting relationships to each other in virtue of having the question of patient responsibility in SDM as a common denominator.

2 Although the patient gains more decision-making power and, as a result, has more responsibility [6], this does not dilute the HCP’s role and responsibility toward the patient [16].

3 This neither asserts nor denies that one is either decision-competent or decision-incompetent regarding a specific decision at a certain point in time. This is because there are relevant practical considerations which influence where one draws the line between capacity and incapacity, e.g., how such a capacity is being assessed [17]. Furthermore, while it is common to understand decision-making capacity as a *threshold* concept, where a person is said to be capable of making a specific decision because her abilities exceed a certain threshold, there is also a more *general* way of understanding decision-making capacity viz. how qualified a person is, generally speaking, to be a decision-maker [20]. Hence, the intention here is not to claim that providing patients with needed opportunities to exercise capacities will necessarily make them *more* capable to make a *specific decision* at a *specific time*, but rather that said opportunities might help patients develop their capacities *in general*. For instance, strength exercises can help one become strong(er) without necessarily implying that she will be able to lift *more* than *X* units of weight, i.e., above a certain threshold.

4 *Control* over one’s actions is a central idea in responsibility/blame literature within philosophy. The intersection of mental illness and moral responsibility is philosophically complex, but it is common to assume that mental illness *often* inescapably affects a person’s capacity for control (to varying degrees), either directly by compromising executive control over one’s actions or indirectly by affecting one’s desires, perceptions, choices among different alternatives etc. which in turn influences actions. Such assumptions can indeed be challenged, whether philosophically or in implications on psychiatric healthcare practices. This paper focuses on the latter.

5 Employing El-Alti et al.’s PCC model [6] and Sandman and Munthe’s [12] SDM levels is not a claim that they are *the* accepted PCC/SDM definitions or to champion them over other definitions or models. However, for the purpose of this paper, the two accounts were chosen for being both grounded in PCC/SDM literature and offering conceptually complex readings of PCC/SDM.

6 From left to right, as illustrated in Fig. 1: Level 1: Patient shares information about herself but HCP makes the decision. Level 2: Patient shares preferences but HCP makes the decision. Level 3: Rational deliberation but HCP makes the decision. Level 8: Rational deliberation followed by joint decision. Level 9: Rational deliberation, conflict, and compromise. Level 7: Rational deliberation but patient makes the decision. Level 6: HCP provides best decision but patient makes the decision. Level 5: HCP helps patient with preferences but patient makes the decision. Level 4: HCP shares relevant info but patient makes the decision (adapted from Sandman & Munthe, 2009) [12].

7 Since there are two parties involved in the SDM process, the responsibility problem is applicable to both of them. However, because this paper is only concerned with patient responsibility, I am assuming that the HCP’s capability to take part in SDM is not under question.

8 The blame aspect of the responsibility problem could still be relevant in non-psychiatric medical settings. However, it might not present a major obstacle as the general expectation is that the patient will uphold the agreements made, consequently bypassing the blame dilemma.

9 Tensions regarding responsibility considerations and ascriptions in PCC are not unique to psychiatry but can also apply to settings like pediatrics and public health, for example. However, this paper is only concerned with psychiatric care contexts.

10 The SDM levels in Fig. 1 are meant to be descriptive of the different possibilities of HCP-patient interactions during decision making, and are neither exhaustive of all the possible interactions between the two parties nor are necessarily indicative of patient limitations at the time of the decision. Which SDM level is chosen can be based on many factors, among which for example, is a person’s (limited) functionality at the time of the decision, but could just as likely be based on the person’s choice, changing needs, or changing lifestyle.

11 The *recovery movement* within psychiatry focuses on a person’s ability to be functional in society[36, 39], and underlines her strengths and resilience instead of deficits [40].

12 I do not assume that Pickard’s account has no objections within the responsibility literature. However, I refrain from delving in them, for two reasons: (1) the literature is voluminous and engaging more substantially with debates on the topic would stray too far from the scope of this paper, and (2) more importantly, the aim of this paper is not to argue *for* Pickard’s account. Even if one objects to (certain parts of) Pickard’s arguments on blameless responsibility, the latter could still be understood instrumentally in the context of this paper, as a way of providing care to a person with mental illness and enhancing her agency while evading the potential harm(s) of blame.

13 These two examples are meant to demonstrate that a *diminished* capacity for responsibility is not the same as a *lack* of capacity for responsibility, and this makes responsibility-sharing possible within SDM in psychiatric contexts. However, the question regarding whether individuals with certain mental illnesses are responsible or blameworthy, in the broad philosophical sense, would be better addressed through moral responsibility (and related philosophical) inquiries.

## Declarations

### Permissions

The author has permission to use the content of the three figures as they appear in the paper.
